# Analyzing the Attenuation of Elastic Waves during Fluid Substitution in Coquina from the Morro do Chaves Formation—A Brazilian Pre-Salt Analogue

**DOI:** 10.1021/acsomega.5c00611

**Published:** 2025-08-19

**Authors:** Simonária Fidelis, Marco Ceia, Roseane Misságia, Lucas Oliveira, Victor Santos

**Affiliations:** † Coordination for the Improvement of Higher Education Personnel (CAPES), Brasília 70040-020, Brazil; ‡ Petroleum Exploration and Engineering Lab (LENEP), 28109 North Fluminense State University (UENF), Macaé 27930-480, Brazil

## Abstract

Understanding seismic attenuation in carbonate rocks is critical for improving reservoir characterization and fluid monitoring during hydrocarbon exploration. This study investigated the behavior of P-wave attenuation (1/*Q*_p_) during fluid substitution from saltwater to oil in coquina samples from the Morro do Chaves Formation, an analogue of Brazilian pre-salt reservoirs. Laboratory experiments were conducted at an ultrasonic frequency (1.3 MHz) by using the spectral ratio method to quantify attenuation. The complex pore structure of coquina, which includes interparticle, moldic, vuggy, and microfracture porosities, significantly influences wave dissipation mechanisms such as squirt flow and Biot-type relaxations. Four samples with different porosity–permeability relationships were analyzed, and attenuation measurements were correlated with saltwater saturation (*S*_w_) under controlled conditions. The results reveal a clear inverse relationship between 1/*Q*_p_ and *S*_w_, with empirical models showing strong correlations (*R* ^2^ > 0.8). Although the experiments were conducted at high frequencies, the observed trends provide insight into frequency-dependent attenuation and fluid mobility effects relevant to field-scale seismic applications. The lack of comparable studies of carbonate rocks with such heterogeneity reinforces the novelty of this work. These findings contribute to our understanding of attenuation mechanisms in porous carbonates and support the development of predictive models for use in reservoir-scale monitoring.

## Graphical abstract

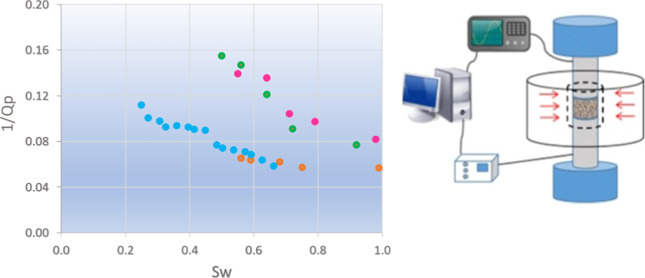

## Introduction

The characterization of carbonate rocks is of significant economic importance to the oil and gas industry, as these formations host some of the world’s largest hydrocarbon reserves.^1^ Understanding the processes of exploration and oil extraction is closely linked to the characterization of fluids within these rocks, during both seismic prospecting and production monitoring. Analyzing petrophysical parameters such as seismic attenuation is essential for optimizing these operations, as it enables the detection of variations in fluid saturation, a key factor in the interpretation of seismic data.^2^ Moreover, carbonate rocks are also used for CO_2_ storage in depleted reservoirs, further emphasizing their relevance in environmental studies.^3^ In this context, attenuation behavior during fluid substitution, a topic of great interest for both hydrocarbon exploration and carbon storage applications, is investigated in this study.

The relationship between the P-wave velocity and the quality factor (or attenuation) plays a crucial role in interpreting subsurface petrophysical parameters, particularly with respect to porosity and permeability.^4^ Seismic attenuation is defined as the progressive loss of wave energy as it travels through a medium and is typically expressed by the inverse quality factor (1/*Q*). This energy dissipation occurs due to a combination of physical mechanisms, including intrinsic absorption (i.e., the conversion of elastic energy into heat due to internal friction), scattering, and interactions related to heterogeneities and the presence of pore fluids.^5,6^ In carbonate rocks in particular, attenuation is strongly influenced by their complex pore architecture, which includes micropores, moldic pores, vugs, and fractures. These features enhance fluid-viscous relaxation and squirt-flow mechanisms, especially in the presence of fluids with contrasting physical properties, such as oil and saltwater.^2,7^

The use of attenuation as a tool in seismic exploration offers several advantages, as it is sensitive not only to the presence of fluids but also to their movement and type within the porous matrix. This allows for the detection of subtle changes in fluid saturation that may not be captured through velocity-based seismic analysis alone. Therefore, attenuation analysis can provide complementary insights into zones of interest, supporting the delineation of productive areas, reservoir characterization, and monitoring of dynamic processes such as CO_2_ injection or fluid replacement in producing reservoirs.^4,6,8^

These parameters are primarily responsible for energy loss mechanisms in porous media, which reinforces the relevance of attenuation studies in seismic exploration.^2^ Previous research on attenuation in rocks with different fluid saturations has focused primarily on sandstones and liquid–gas or liquid–air systems.^9–11^ However, the literature on attenuation in carbonate rocks, particularly concerning fluid substitution, is still limited, highlighting the importance of this study, which investigates the effect of fluid replacement in coquina samples from the Morro do Chaves Formation in Brazil.^5^

The coquinal rocks of the Morro do Chaves Formation, located in the Sergipe-Alagoas Basin, present a highly complex porous framework that is primarily composed of shell fragments and other bioclastic debris. These characteristics result in variable porosity and permeability, making them challenging porous systems to characterize.^12,13^ In addition, the heterogeneity of these rocks contributes to the complexity of their seismic response, requiring a detailed investigation of how processes such as fluid substitution can influence their physical properties and seismic behavior. In this sense, the Morro do Chaves Formation serves as an important analogue for coquina reservoirs in the Brazilian presalt region, particularly in studies focused on the effects of fluid saturation changes on seismic attenuation.

Previous studies on attenuation in rocks with different fluid saturations have focused mainly on sandstones and liquid–gas or liquid–air systems.^9,10^ However, the lack of specific data on attenuation in carbonate rocks, especially with respect to fluid substitution, underscores the novelty of this research. The coquina rocks from the Morro do Chaves Formation, with their intricate pore structures, are comparable to those found in the pre-salt reservoirs, a region of great interest to the petroleum industry. However, experimental studies on attenuation in pre-salt analogue rocks, particularly with respect to fluid replacement, remain virtually nonexistent, which constitutes a significant gap in the literature.^5^ This study aims to fill that gap by using saltwater and oil as saturating fluids to simulate reservoir conditions and investigate the relationship between attenuation and fluid substitution in carbonate rocks.

With the goal of advancing our understanding of attenuation in carbonate systems, especially under fluid substitution scenarios, this work adopts an unprecedented experimental approach. The analysis of attenuation behavior in coquina rocks saturated with different fluids not only enhances our understanding of their physical properties but also supports the development of empirical models capable of predicting the effects of fluid saturation and substitution on seismic attenuation. The experimental results presented in this study offer valuable insights for the oil and gas industry by providing a new perspective on carbonate reservoir characterization and optimization of exploration processes.^6,14^

## Theory

### Attenuation

The concept of seismic attenuation involves measuring the relative decay of mechanical energy stored during an oscillation process. This energy decay can be observed as a spatial logarithmic decrement of amplitude for a traveling wave or as a broadening of a spectral peak and/or a strain–stress phase lag in a stationary (subresonant) forced-oscillation test conducted on a rock sample in the laboratory.^15^

Among the various methods for estimating and describing attenuation (α\alphaα), the most commonly used method is related to the exponential decay of the amplitude of a plane wave.^16^ The amplitude of plane-wave propagation in an elastic homogeneous medium is expressed by the following equation:

1 $$A\left(x,t\right)=A_{0}e^{i\left(kx-\omega t\right)}$$

where *A*_0_ is the maximum value of the amplitude, ω is the frequency, *k* is the wavenumber, *t* is the time, and *x* is the position. The equation below shows the relationship between a complex wavenumber and attenuation.

2 $$k=K_{r}+i\alpha$$

where *K*_r_ is the real part of the wavenumber, α is the plane-wave attenuation coefficient, the units are the inverse length, and the plane-wave equation becomes^16^

3 $$A\left(x,t\right)=A_{0}e^{-\alpha x}e^{i\left(K_{r}x-\omega t\right)}$$

Given the definition of attenuation described above, one can define α in terms of the amplitude of the wave at two different positions *x*_1_ and *x*_2_.^16,17^

4 $$\alpha =\frac{1}{\left(x_{2}-x_{1}\right)}\ln\left[\frac{A\left(x_{1}\right)}{A\left(x_{2}\right)}\right]$$

It can also be written in this way:

5 $$\alpha =\frac{1}{\left(x_{2}-x_{1}\right)}20\;\mathrm{Log}\left[\frac{A\left(x_{1}\right)}{A\left(x_{2}\right)}\right]$$

where α is the attenuation exponent and is expressed in dB m^–1^ or nepers m^–1^, the frequently used unit for dB/m (decibel/m).

The conversion between the two measures is as follows:

$$\alpha \;\left(in\;dB\;m^{-1}\right)=8.686\times \alpha \;\left(in\;nepers\;m^{-1}\;\text{or}\;m^{-1}\right)$$

$$\alpha \;\left(in\;nepers\;m^{-1}\;\text{or}\;m^{-1}\right)=0.115\times \alpha \;\left(in\;dB\;m^{-1}\right)$$

The quality factor (*Q*) or its inverse *Q* ^–1^ is a measure of how dissipative the material is. Small changes in the phase velocity can be ignored when the low-loss assumption *Q* ^–1^ can be expressed by the following equation:^17^

6 $$Q^{-1}=\alpha \frac{V}{\pi f}$$

where *V* is the velocity and *f* is the frequency.

For viscoelastic media, in general, the following equation is used:^17^

7 $$Q^{-1}=\alpha \frac{V}{\pi f-\frac{\alpha^{2}V^{2}}{4\pi f}}$$

where *Q* ^–1^ represents the attenuation normalized by the wavelength.

If α is a linear function of frequency in the first approximation, then *Q* ^–1^ is independent of frequency; in this way, it can describe the attenuation properties of rocks without a frequency consideration.

### Attenuation Mechanisms

Seismic attenuation in porous media refers to the loss of energy associated with the propagation of elastic waves, which primarily occurs through the conversion of mechanical energy into heat. This energy dissipation can be caused by different physical mechanisms that act on various spatial scales and is heavily dependent on the properties of the rock and the saturating fluid. Among the main attenuation mechanisms recognized in the literature are Biot’s inertial flow, local or squirt flow, patchy saturation, and scattering due to heterogeneities.

### Biot’s Inertial Flow

The model developed by Biot^18^ forms the foundation of the theory of wave propagation in fluid-saturated porous media. This mechanism accounts for the relative motion between the solid matrix and the pore fluid as a source of energy dissipation caused by viscous drag. The effectiveness of this dissipation depends on the wave frequency, porosity, permeability, fluid viscosity, and fluid density. Notably, when the wave frequency approaches Biot’s critical frequency, attenuation increases significantly.

According to Mavko et al.,^5^ this mechanism is particularly relevant in the ultrasonic frequency range (above 100 kHz) for rocks with intermediate to high permeability. In coquina rocks, Biot’s attenuation may become significant when the porosity and permeability values fall within the critical range, as shown in various laboratory studies.

### Squirt Flow

Squirt flow refers to the dissipation of energy caused by fluid motion between pores of different sizes (e.g., from microcracks to intergranular pores) during the application of an elastic wave. This mechanism is highly sensitive to the pore geometry, especially the aspect ratio, and to the compressibility of the solid mineral. It operates on microscopic scales and is generally active at relatively high frequencies, often in the ultrasonic range.

Recent studies, such as those by Gurevich et al.^19^ and Alkhimenkov and Quintal,^20^ have demonstrated that squirt flow is one of the main contributors to the wave velocity dispersion and attenuation in heterogeneous carbonate rocks. Its importance increases in rocks with significant microporosity and well-connected pore networks.

### Patchy Saturation

The patchy saturation mechanism, proposed by White et al.,^21^ considers the presence of regions within a porous medium that are saturated with different fluids (e.g., water and gas) at mesoscopic scales. Differences in fluid compressibility generate pore pressure imbalances during wave propagation, which are relieved through local fluid flows, thereby causing energy dissipation.

This mechanism is most efficient at low frequencies (tens to hundreds of Hz), particularly when gas is present or during partial fluid substitution. Although it is typically not dominant at ultrasonic frequencies, it must be considered in studies involving partial saturation and fluid replacement, as noted by Pride et al.,^22^ Rubino et al.,^23^ and Chapman et al.^24^

## Experimental Techniques

### Gas Porosimeter

The measurements using an UltraPore 300 gas porosimeter are based on Boyle’s law (also known as the Boyle–Mariotte law), which describes the relationships among the volume (*V*), pressure (*P*), and temperature (*T*) of a gas, such as helium or compressed air. These relationships are expressed by the following formula:

8 $$\frac{P_{i}V_{i}}{T_{i}}=\frac{P_{f}V_{f}}{T_{f}}$$

where i = initial and f = final.

The gas porosimeter operates with a graphical interface provided by WINPORE software, which requires the sample’s weight, diameter, and length as input data. On the basis of this information, the software calculates and outputs the bulk volume, grain density, and effective porosity of the sample.

Additionally, the grain volume (gr_v_) can be determined by taking the difference between the total volume and the pore volume, providing a more complete understanding of the sample’s internal characteristics.

9 $${gr}_{v}=T_{v}-P_{v}$$

where *T*_v_ represents the total volume of the sample and *P*_v_ denotes the pore volume.

### Measurement of Acoustic Velocity

Using the rock physics system equipment (Figure 1) located in the Deformation and Physical Properties Laboratory of Rocks at LENEP/UENF, it is possible to measure the acoustic wave velocities in rock samples.

**Figure 1:**
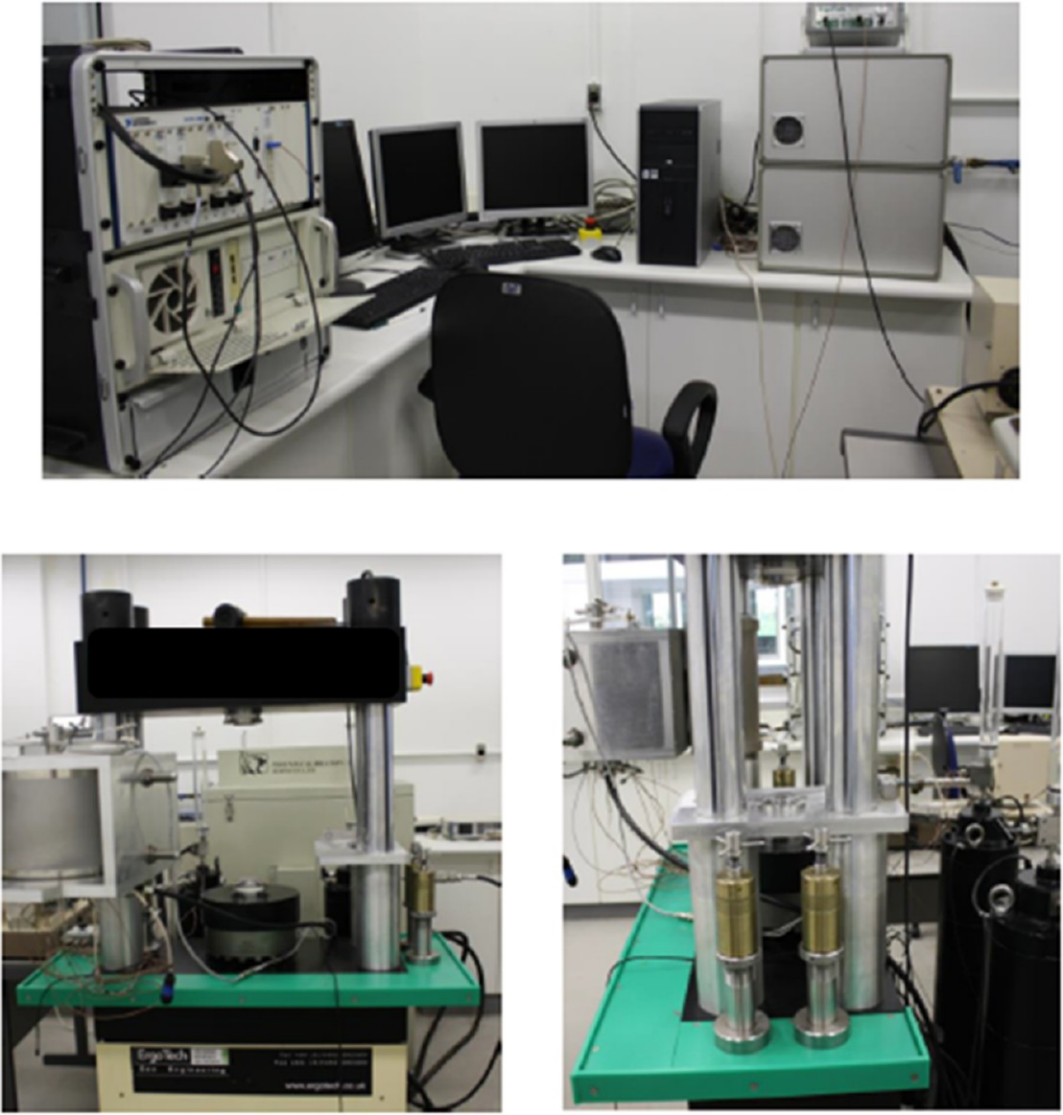
Rock physics system equipment.

The pulse transmission method is used to calculate wave velocities at approximately 1.3 MHz. This is achieved by measuring the transit time of a high-frequency elastic pulse as it propagates through the sample. The pulse signal is displayed on an oscilloscope, which recorded the total transit time through both the system and the sample. The propagation velocity of the elastic waves is then determined via the following formula:

10 $$V=\frac{\Delta x}{\Delta t}$$

where Δ*x* represents the sample length and Δ*t* represents the measured transit time of the wave through the sample.

The time taken by the wave to travel through the sample (Δ*t*) is equal to the observed delay time (Δ*t* = *t* – *t*_0_) recorded on the oscilloscope, where *t*_0_ represents the time delay caused by the signal’s travel time through the electronics and metal heads when no sample is present in the system. This delay is determined through direct contact tests when the transducers are positioned face-to-face.

To enhance contact between the transducer and improve transmission of the ultrasonic wave, lead foils were placed at the edges of the samples. The waveforms P, S1, and S2 are acquired for each sample and correspond to the *Z*-axis (vertical direction). PicoScope software is utilized to display and capture these waveforms, enabling time analysis, as well. The amplitude of each wave is analyzed as a function of the recorded transit time.

To determine the velocity, the traditional method involves measuring the time of a specific event, such as the first break or peak of the first wave. In the literature, estimating the transit time by recording the first break is the most commonly used method.

### Gas Permeability

The permeability of the samples was measured via an axial flow of nitrogen gas with a PERG-200 gas permeameter and a core holder to apply radial confining pressure to the samples. This setup allowed for the determination of flow values at various applied pressures. The operational procedures followed the guidelines set by API.^25^

To estimate the absolute permeability, Darcy’s law (eq 11) was employed. By plotting the relationship between the flow rate, *Q*, and the pressure difference (*P*_i_^2^ – *P*_o_^2^) over the mean pressure, *P*_m_, a linear fit was generated. The slope of this fit provides the permeability (*k*) value. The permeability values obtained with PERG-200 ranged from approximately 1 to 2000 mD.

11 $$k=2000\frac{L}{A}\mu Q\frac{P_{m}}{\left(P_{i}^{2}-P_{o}^{2}\right)}$$

### X-ray Diffractometry (XRD)

According to Albers et al.,^26^ XRD is a technique used to evaluate the crystalline phases of a given material. XRD enables qualitative and quantitative identification of components in mineralogical crystal samples.

To identify these components, it is essential that Bragg’s law be satisfied. When Bragg’s law holds true, constructive interference occurs; conversely, when it is not satisfied, destructive interference arises, and no signal is observed.

A crystal consists of atomic planes spaced at equal distances (*d*). When a crystal is illuminated with a plane wave of wavelength (λ), diffraction takes place. Constructive interference occurs when the path length difference between the incident ray and the rays diffracted by the atoms equals an integer multiple of the wavelength. The angle 2θ is defined as the angle between the direction of the incident ray and the direction of the diffracted rays.

This phenomenon leads to Bragg’s law.

12 nλ=2dsin⁡θ

### X-ray Fluorescence (XRF) Spectrometry

The XRF method was employed to determine the chemical composition of the materials. In this technique, X-ray radiation is emitted onto the sample, leading to interactions between the sample’s elements and the incoming X-rays. As a result, fluorescence radiation is emitted with energies characteristic of each chemical present in the sample (Figure 2).

**Figure 2:**
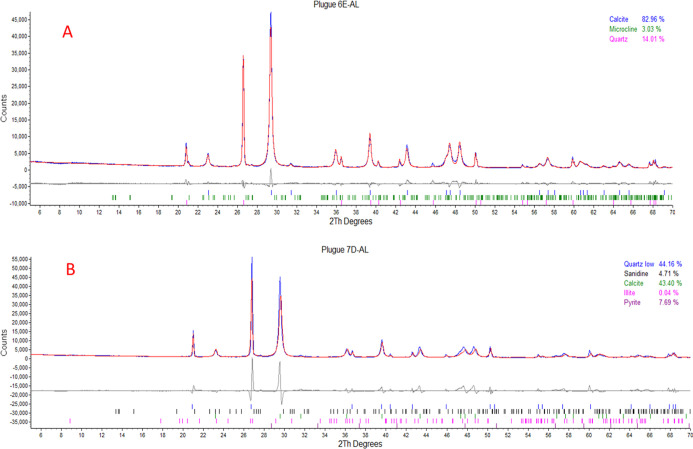
Example diffractograms of samples 6E-AL (A) and 7D-AL (B).

Qualitative analysis is performed by examining the energy peaks corresponding to the elements, whereas quantitative analysis is conducted by measuring the intensity of each peak. This method is particularly useful for confirming XRD results by comparing the mineral constituents and the chemical elements identified through XRF.

### Geological Overview

Four samples were analyzed to study the behavior of attenuation during the fluid replacement process: 7D-AL, 6E-AL, 9C-AL, and 4.3B-AL. These samples are carbonate rocks known as coquina, sourced from the Morro do Chaves Formation in the Sergipe-Alagoas Basin, located on the eastern coast of Brazil, encompassing the states of Sergipe, Alagoas, and Pernambuco. The formation has an elongated shape trending in the NE/SW direction (Figure 3).

**Figure 3:**
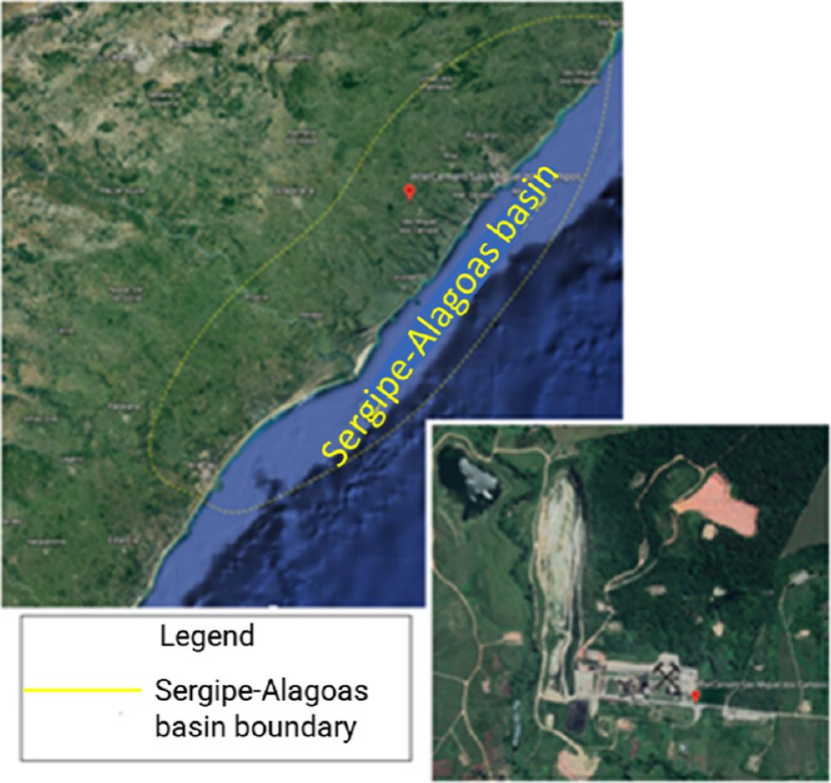
Location map of the Sergipe-Alagoas Basin. Satellite image from Google Maps. Map data ©2025 Google, IBGE, CNES/Airbus. Reproduced in accordance with Google Maps Terms of Service.

As reported by Filho et al.,^27^ the Morro do Chaves Member indicates a return to a lacustrine environment, marked by the influx of terrigenous alluvial fans interspersed with pelitic and carbonate lacustrine sediments. Variations in lake levels due to subsidence partially inundated the terrigenous sediments, leading to the deposition of coquinas and pelites. The shallow, oxygenated waters favored the development of bivalves, whose shells were subsequently reworked by storm waves and accumulated at the lake bottom, forming coquinas. The pelites, on the other hand, originated during periods of maximum lake levels and anoxic conditions, which helped preserve the organic matter.

## Methods

### Spectral Ratio Method

The attenuation coefficient (α) was determined via the spectral ratio method, which assumed that the attenuation coefficient is a linear function of frequency. This method measures attenuation, or its inverse *Q*, by comparing two recorded waves: one from the rock sample and the other from a reference sample, both of which have the same geometry. The reference sample must consist of a material with negligible attenuation.^28^ Accordingly, as stated by Toksöz et al.,^28^ the amplitudes of plane seismic waves for both the reference and the sample can be expressed as

13 $$A1\left(f\right)=G1\left(x\right)e^{-\alpha 1\left(f\right)x}e^{i\left(2\Pi ft-k1x\right)}$$

and

14 $$A2\left(f\right)=G2\left(x\right)e^{-\alpha 2\left(f\right)x}e^{i\left(2\Pi ft-k2x\right)}$$

where *A* = amplitude, *f* = frequency, π ≅ 3.1415, *k* = 2Π*f*/*v* = wavenumber, *x* = distance, *V* = velocity, *G*(*x*) = a geometrical factor that includes spreading, reflections, etc., and α­(*f*) is the frequency-dependent attenuation coefficient. In the range of frequencies used in the laboratory (tens to hundreds of kHz), the attenuation coefficient α (*f*) varies linearly with frequency; thus, one can write

15 α(f)=γf

where γ is a constant related to the quality factor *Q* as follows:

16 $$Q=\frac{\Pi }{\gamma v}$$

When the same geometry is used for both the sample and the standard, *G*1 and *G*2 are frequency-independent scale factors. The ratio of the Fourier amplitudes is as follows:

17 $$\ln\left(\frac{A1}{A2}\right)=\left(\gamma 2-\gamma 1\right)xf+\ln\left(\frac{G1}{G2}\right)$$

where *x* is the sample length.

Since *G*1/*G*2 is independent of frequency, the difference (γ2 – γ1) can be obtained from the slope of the natural logarithm of the spectral ratio of the amplitudes plotted against frequency. Given that the *Q* factor of the reference sample is very high, the rock attenuation coefficient can be directly determined from this slope.

### Extraction of Attenuation Attributes

To obtain the attenuation coefficient and quality factor of the samples via the spectral ratio method, a program was developed in MATLAB on the basis of the script by Marques.^29^ The reference sample used was aluminum, which was selected for its negligible attenuation properties.

Initially, the program imports the waveforms of the reference sample and rock samples. The next step involves selecting the portion of interest from the waveforms for the calculation of the attenuation attributes. To identify this segment of the P-wave, we first determined the time corresponding to the first break and then selected a wave train on the basis of this reference point.

Once the area of interest is identified, a high-pass filter is applied to process both the aluminum and rock waveforms. The linear interpolation method is then employed to relate the logarithm of the amplitude ratio of the reference wave to the rock wave versus the frequency.

Finally, using this relationship, the attenuation coefficient and quality factor are calculated as outlined in eqs 12–16. The program outputs several graphs, including (1) the normalized amplitude versus frequency (for both the reference and rock samples) and (2) the natural logarithm of the amplitude ratio between the reference cylinder and the sample versus frequency (see Figure 4). This figure includes a fitted linear regression, which is used to estimate the attenuation coefficient and quality factor.

**Figure 4:**
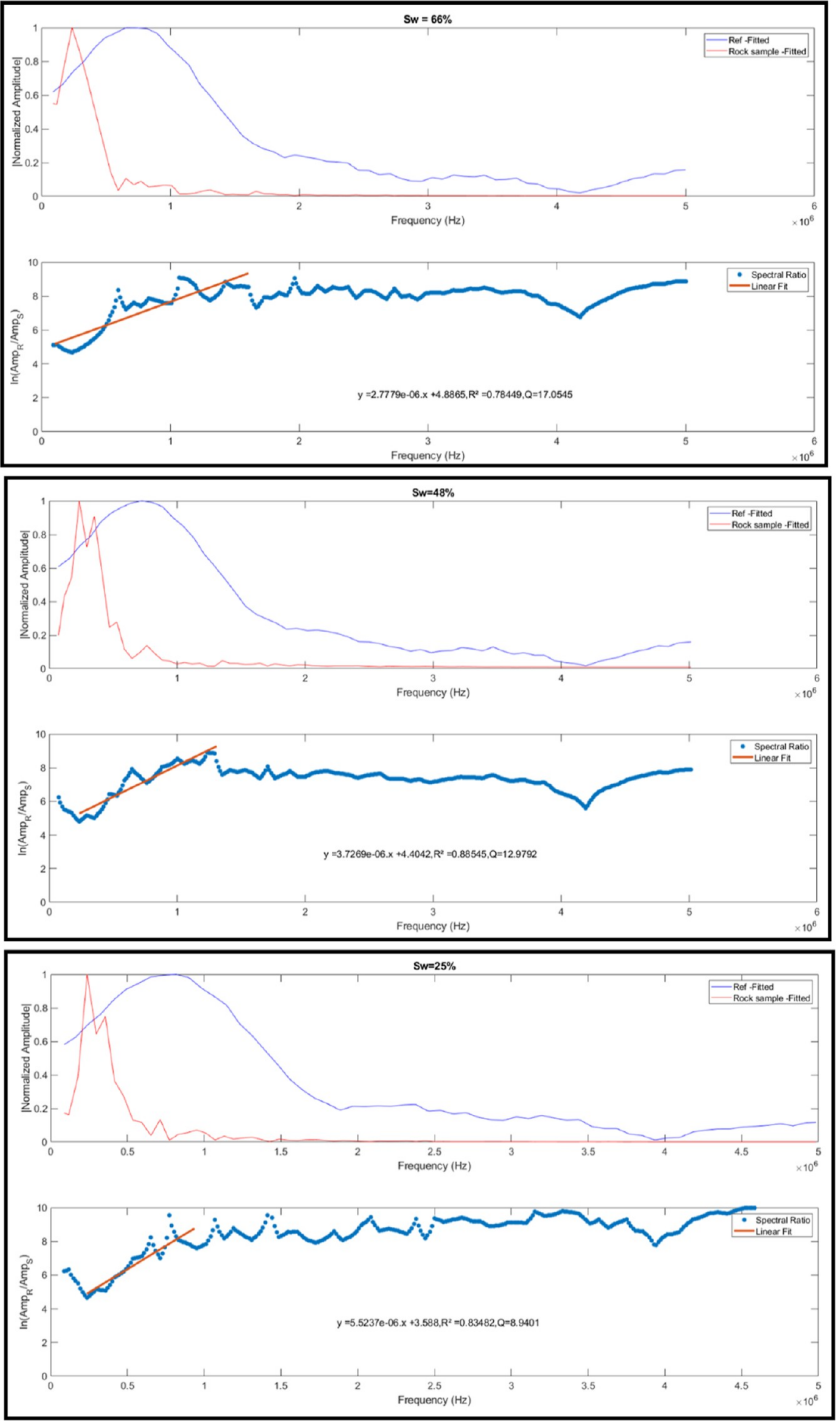
Graphs of normalized amplitudes versus frequency (top) and the natural logarithm of the ratio between the amplitudes of the reference wave and rock sample 4.3B-AL (saturated with saltwater at 50,000 ppm of NaCl). These graphs are presented for 3 different *S*_w_ values. The red line represents the linear fit of the logarithm of the ratio between amplitudes.

### Window Length and Frequency Range Selection

The selection of the time window and frequency range used in the spectral ratio method was based on preliminary tests performed with the waveforms of both the rock samples and the reference sample (aluminum). These tests aimed to maximize the signal-to-noise ratio and ensure that the selected segment captured the most representative portion of the P-wave energy, particularly within the interval corresponding to the first arrivals while avoiding overlap with multiple events or background noise.

The time window was defined by identifying the first break, followed by visual inspection of the signal segment that exhibited stable amplitude behavior characteristic of the P-wave. To maintain consistency across analyses, a fixed window relative to the first arrival was applied to all samples.

The frequency range was determined through spectral analysis via fast Fourier transform, which was applied to both the sample and reference waveforms. The selected band corresponded to the frequency interval common to both spectra with a high energy content and minimal noise interference. Additionally, the chosen range avoided edge effects and ensured a reliable linear fit in the logarithmic spectral ratio plot, a necessary condition for the proper application of the method.

These methods are essential for ensuring the reliability of the attenuation coefficients (α) and quality factors (*Q*_p_) obtained, supporting the reproducibility of the results and enabling comparisons between the different saturation levels of the analyzed samples.

### Experimental Apparatus

To replace liquid fluids within the rock sample, the triaxial rock deformation system was connected to an apparatus, as shown in Figure 5. This apparatus (Figure 5A) consists of a positive displacement pump that maintains a constant flow and pressure, a fluid transfer bottle, a pressure gauge, hydraulic pipes and connections, and a beaker for fluid collection and system relief.

**Figure 5:**
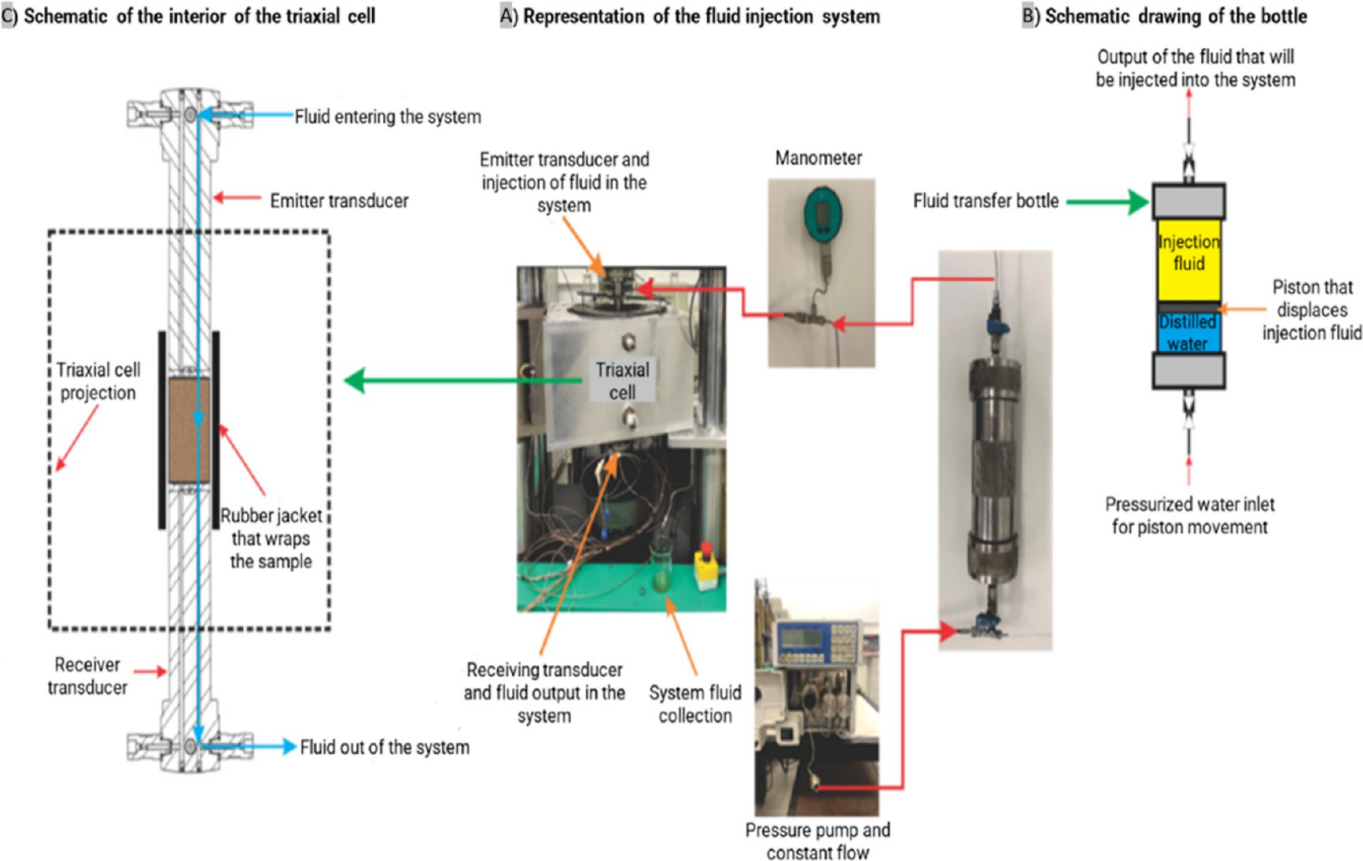
Overview of the triaxial rock deformation system coupled to the apparatus.

The tests begin with the progressive application of axial and radial loads up to 40 MPa, assuming that the sample is already saturated with saltwater. Following this, the fluid injection process commences. A pump with constant pressure and flow (at a rate of 10 mL/min) draws distilled water from a reservoir and injects it into the lower chamber of the transfer bottle. This generates sufficient pressure to push the plunger, displacing the fluid from the second chamber into the triaxial cell (Figure 5B). The transfer bottle is connected to an ultrasonic transducer at the top, facilitating communication with the sample in the triaxial cell and enabling fluid collection at the bottom transducer outlet (as shown in Figure 5C).

During the fluid replacement process, the triaxial rock deformation system takes ultrasonic measurements of P- and S-waves, which are later used to calculate the attenuation.

To ensure the proper functioning of the experiment and eliminate any fluids that could interfere with the injection process (such as air or residual water in the injection lines), the system must be drained by an open-system injection. After the injected fluid passes through the entire system, it is collected in a beaker positioned at the outlet of the lower transducer, until the collected fluid becomes homogeneous and free of impurities or air bubbles.

## Results and Discussion

### Geological Characterization

Four samples (6E-AL, 7D-AL, 9C-AL, and 4.3B-AL) were analyzed via petrographic images (Figure 6) to characterize their internal structures on the basis of Dunham’s classification^30^ and pore-type classification via Choquette and Pray.^30^ The mineralogical composition was also examined through XRD and XRF analyses (Table 1).

**Figure 6:**
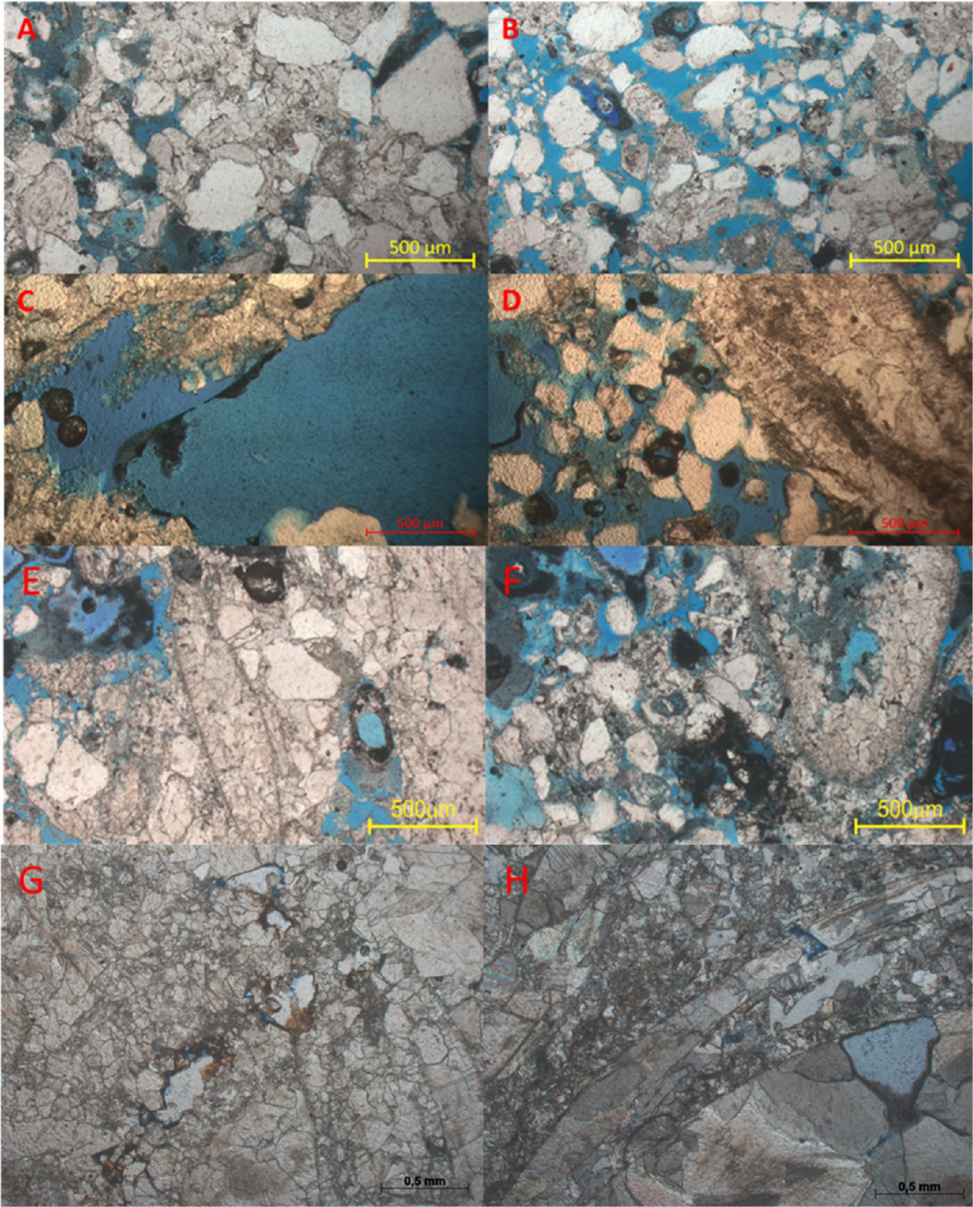
Thin sections of samples 6E-AL (A,B), 7D-AL (C,D), 9C-AL (E,F), and 4.3B-AL (G,H) reveal various internal structures and porosity types. For sample 6E-AL, (A) shows mono- and polycrystalline quartz grains, whereas (B) displays interparticle porosity. For sample 7D-AL, (C) highlights megapore vugular porosity, and (D) shows interparticle porosity between quartz grains and bivalve clasts. In sample 9C-AL (E,F), a grainstone-type depositional texture is evident, with vugular porosity as the most common type and the presence of isopachous fringe-type cement along the edges of the bioclasts. For sample 4.3B-AL (G,H), the primary porosity is predominantly vuggy and moldic, and the shells are filled with blocky sparry calcite cement ranging from microcrystalline to macrocrystalline. A bivalve clast with a relict internal structure and neomorphic sparry calcite cement (originally aragonitic in composition) was observed. The presence of drusy calcite cement surrounding the edges of some bivalve clasts was noted, along with ferruginous cement forming a black to brownish crust that filled the pores.

**Table 1:** Geological Descriptions of Sample 6E-AL

sample	mineralogy	porosity (%)	classification by Dunham^31^	pore type—Lucia^32^
6E-AL	calcite (82.96%), quartz (14.01%), microcline (3.01%)	18.93 ± 0.003	grainstone	interparticle-subordinate fracture
7D-AL	calcite (43.4%), quartz (44.16%), sanidine, (4.71%), pyrite (7.69%), illite (0.04%)	17.27 ± 0.004	grainstone	interparticle and vugular
9C-AL	calcite (80.73%), quartz (10.39%), sanidine (7.13%), marcasite (1.75%)	15.42 ± 0.0004	rudstone	interparticle
4.3B-AL	calcite (98.27%), quartz (1.73%)	15.33 ± 0.002	rudstone	moldic

### Saltwater and Oil Preparation and Saturation of Rock Samples

To prepare a sample for initial saturation, it first undergoes a drying process to remove any residual moisture absorbed during the plug production process. Drying is carried out in a conventional oven at 60 °C for at least 12 h. Once dried, the sample is immersed in saltwater and placed in a vacuum desiccator to achieve complete saturation. The saltwater has a concentration of 50,000 ppm of NaCl and a density of 1.0403 g/cm^3^.

Following saturation with saltwater, the sample is subjected to a fluid replacement process, in which the second fluid introduced is an oil mixture designed to simulate an oil with an API gravity of 25–26° API. This mixture is prepared by combining synthetic oil and diesel oil. Initially, the densities of both synthetic oil and diesel oil were measured via a pycnometer, after which the API gravity for each was calculated, as shown in Table 2.

**Table 2:** Characterization of Synthetic and Diesel Oil

type of oil	density (g/cm^3^)	API degree
diesel	0.9657	15
synthetic	0.7582	55

To achieve the desired API gravity, a mixture was prepared using 25 mL of synthetic oil for every 50 mL of diesel oil, resulting in an API gravity of 26. Additionally, a viscosity analysis of this mixture was conducted, as shown in Table 3.

**Table 3:** Characterization of the Oil Mixture

oil mixture	
density (g/cm^3^)	0.8965
API grade	26
viscosity (cP)	13.83

### Porosity and Permeability Results

Porosity and permeability tests were conducted on samples 6E-AL (length: 6.28 cm, diameter: 3.80 cm), 7D-AL (length: 6.99 cm, diameter: 3.79 cm), 9C-AL (length: 6.99 cm, diameter: 3.79 cm), and 4.3B-AL (length: 7.45 cm, diameter: 3.76 cm).

Table 4 presents the porosity and permeability results obtained without confining pressure via the UltraPore 300 and PERG-200 instruments.

**Table 4:** Results for Porosity and Permeability

sample	porosity (%)	permeability (mD)
6E-AL	18.93 ± 0.003	212.18 ± 0.59
7D-AL	17.27 ± 0.004	221.01 ± 0.59
9C-AL	15.42 ± 0.0004	178.88 ± 0.59
4.3B-AL	15.33 ± 0.002	155.66 ± 0.59

### Velocities and Attenuation Results

To determine the P-wave velocity, the arrival time corresponding to the first break was identified in accordance with ASTM^33^ standard. The experimental uncertainty in velocity measurements was approximately 1%, based on calibration tests conducted using rock physics laboratory equipment, as reported by Lima Neto et al.^34^ To improve the signal-to-noise ratio and facilitate accurate identification of the first arrival, a band-pass filter was applied to the recorded waveforms. This filter type selectively transmits frequency components within a defined range while attenuating both low-frequency background noise and high-frequency undesired components. As a result, the spectral content of the most representative part of the P-wave signal was isolated. Figure 7 illustrates the application of this criterion, with the vertical dashed line indicating the arrival time used in the velocity analysis.

**Figure 7:**
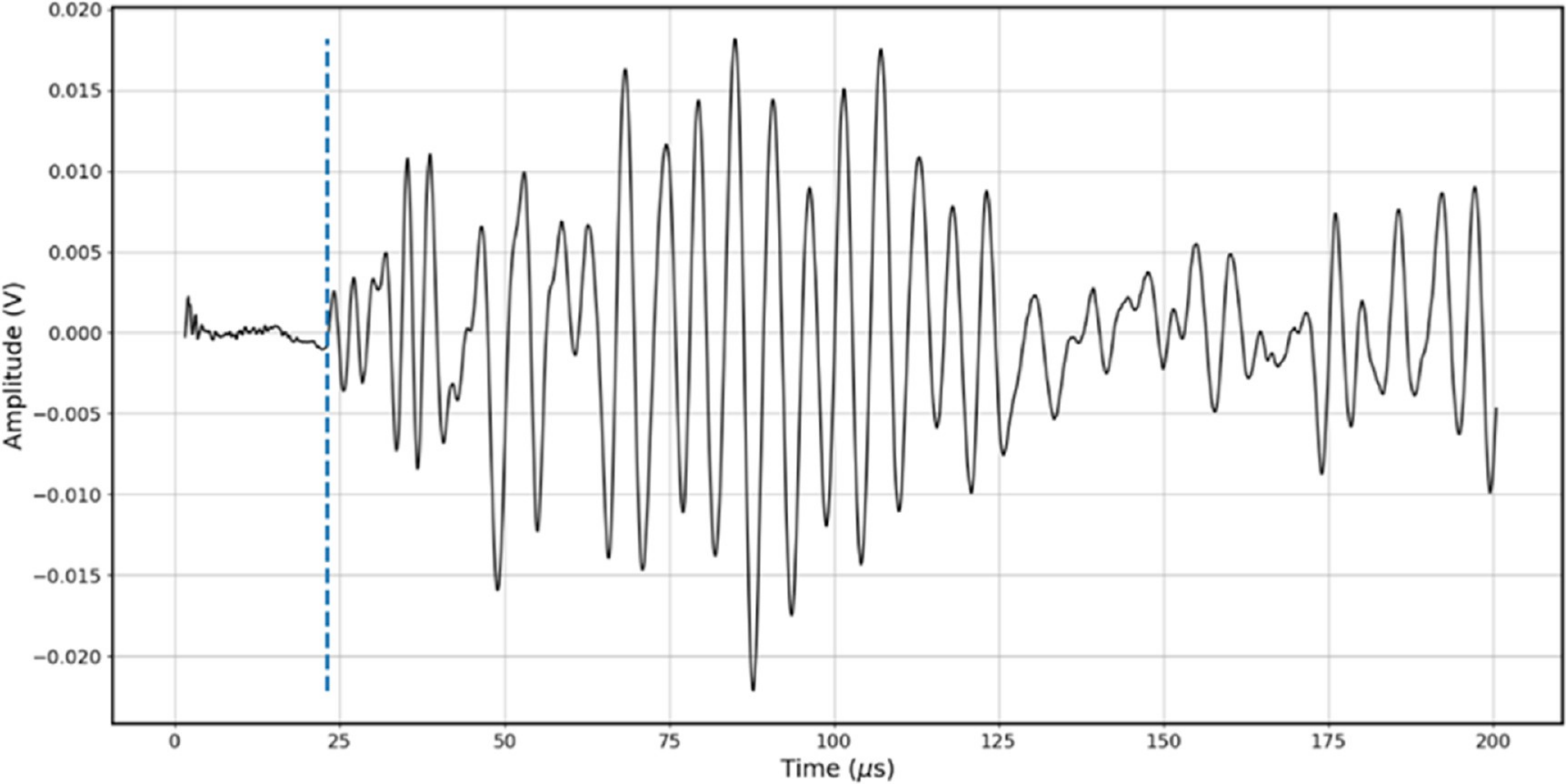
Graph showing the determination of the P-wave arrival time for the 4.3B-AL sample fully saturated with saltwater (50,000 ppm of NaCl), under an effective pressure of 19.75 MPa.

The propagation P wave velocity (km/s) is calculated as follows:

18 $$V_{p}=\frac{L}{T_{p}}$$

where *L* is the vertical length of the sample, [mm], and *T*_p_ represents the effective transit time (measured time minus the correction time *t*_0_) of the P wave, [μs].

The samples that were previously saturated with saltwater were subsequently filled with the second fluid, resulting in automatic expulsion of the saltwater. The effective pressure applied during this experiment was 19.75 MPa. This second fluid, a mixture of hydraulic oil and diesel oil, has an API gravity of 26, a density of 0.89 g/cm^3^, and a viscosity of 13.83 cP under reference conditions (25 °C).

It is anticipated that attenuation measurements could predict this fluid change. Table 5 presents the results for the P-wave velocity, attenuation coefficient, and quality factor following fluid replacement. The error in the *Q* factor was calculated on the basis of the coefficient of determination of the linear fit between the ln (normalized amplitude) and frequency.

**Table 5:** Results of P-Wave Velocity, Attenuation Coefficient, and P-Wave Quality Factor (α_p_, *Q*_p_) Concerning Saltwater Saturation (*S*_w_)

sample	*S*_w_ (%)	*V*_p_ (km/s)	α_p_ (dBm^–1^)	*Q*_p_
7D-AL	0.9800	4.7309 ± 0.047	0.00005442	12.20 ± 0.73
	0.7900	4.7250 ± 0.047	0.00006496	10.23 ± 0.51
	0.7100	4.7023 ± 0.047	0.00006970	9.59 ± 0.28
	0.6400	4.6960 ± 0.046	0.00009076	7.37 ± 0.44
	0.5500	4.5702 ± 0.045	0.00009582	7.17 ± 0.43
6E-AL	0.9200	4.3881 ± 0.043	0.00005290	12.99 ± 0.38
	0.7200	4.3336 ± 0.043	0.00006267	10.97 ± 0.21
	0.6400	4.3127 ± 0.043	0.00008336	8.25 ± 0.24
	0.5600	4.2804 ± 0.042	0.00010096	6.81 ± 0.27
	0.5000	4.0933 ± 0.040	0.00010663	6.45 ± 0.45
9C-AL	0.9900	4.593 ± 0.045	0.00003899	17.63 ± 0.17
	0.7500	4.557 ± 0.045	0.00003931	17.48 ± 0.52
	0.6800	4.519 ± 0.045	0.00004279	16.06 ± 0.48
	0.5900	4.504 ± 0.045	0.00004387	15.67 ± 0.31
	0.5600	4.458 ± 0.044	0.00004501	15.27 ± 0.61
4.3B-AL	0.8840	4.978 ± 0.049	0.000034709	18.18 ± 3.09
	0.6620	4.945 ± 0.049	0.00003725	17.05 ± 4.09
	0.6265	4.926 ± 0.049	0.00004065	15.69 ± 3.45
	0.5910	4.922 ± 0.049	0.00004367	14.62 ± 2.63
	0.5733	4.900 ± 0.049	0.00004557	14.07 ± 2.67
	0.5377	4.871 ± 0.048	0.00004687	13.76 ± 1.92
	0.5022	4.849 ± 0.048	0.00004803	13.49 ± 1.75
	0.4845	4.843 ± 0.048	0.00004998	12.98 ± 2.20
	0.4490	4.836 ± 0.048	0.00005833	11.14 ± 1.33
	0.4134	4.830 ± 0.048	0.00005889	11.05 ± 0.88
	0.3957	4.827 ± 0.048	0.00006025	10.80 ± 0.75
	0.3602	4.796 ± 0.047	0.00006146	10.66 ± 1.49
	0.3247	4.790 ± 0.047	0.00006082	10.78 ± 2.37
	0.3069	4.774 ± 0.047	0.00006441	10.22 ± 1.83
	0.2714	4.771 ± 0.047	0.00006643	9.91 ± 2.18
	0.2500	4.744 ± 0.047	0.00007407	8.94 ± 0.44

To assess the influence of the internal structure of the samples on seismic attenuation, attenuation measurements were conducted on samples fully saturated with saltwater. This approach allows the isolation of the effects of porosity, pore connectivity, and the presence of fractures on the dissipation of seismic wave energy, ensuring that the observed attenuation is predominantly associated with the intrinsic characteristics of the rock.

Figure 8 presents a scatter plot relating the porosity (%) on the *x*-axis and the P-wave quality factor (*Q*_p_) on the *y*-axis. The color of the data points is controlled by the calcite percentage, as indicated by the color scale on the right side of the image. The data are distributed across four points, highlighting significant variations in both porosity and *Q*_p_, as well as clear mineralogical differentiation on the basis of coloration.

**Figure 8:**
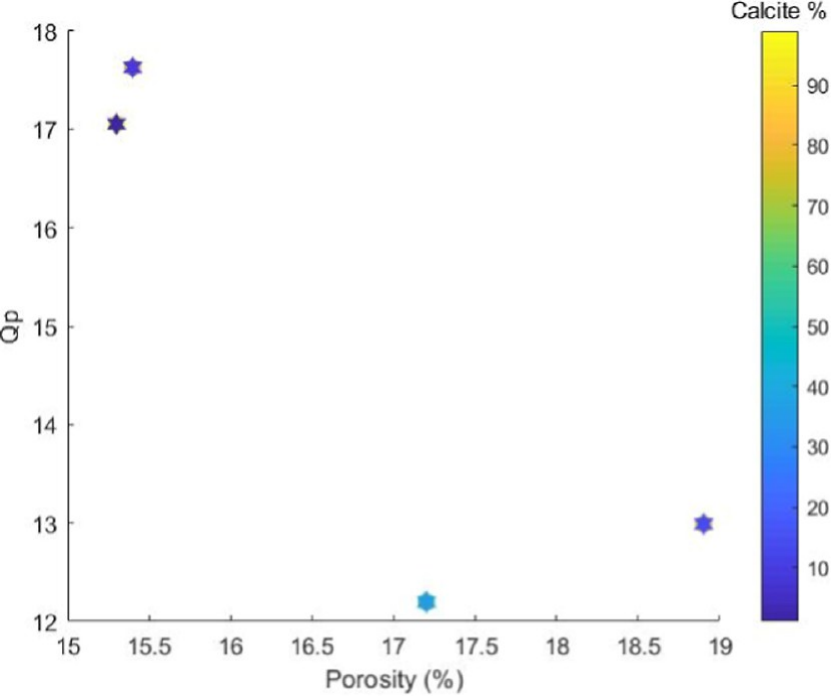
Graph showing a scatter plot relating porosity (%) on the *x*-axis and the P-wave quality factor (*Q*_p_) on the *y*-axis. The color of the data points is controlled by the calcite percentage.

The points located in the region of lower porosity (∼15.5%) present the highest *Q*_p_ values (∼17–18), suggesting lower seismic energy dissipation in these samples. This behavior may be associated with a more compact and mechanically rigid rock matrix, possibly due to the lower pore connectivity and the predominance of calcite, a mineral with high density and a high elastic modulus. Previous studies indicate that, in carbonate rocks, porosity and mineralogy strongly influence seismic attenuation, as the presence of interconnected pores and microfractures facilitates energy dissipation mechanisms (Dvorkin and Nur,^8^ Eberli et al.^35^). Additionally, Carcione et al.^36^ highlight that the attenuation of elastic waves is amplified by the presence of mineralogical heterogeneities and fluids in the pores, reinforcing the hypothesis that denser structures exhibit lower energy dissipation.

On the other hand, the point with intermediate porosity (∼17%) displays the lowest *Q*_p_ value (∼12.5), which coincides with light blue coloration and represents a reduced calcite content (∼20%). These results suggest that samples with lower calcite concentrations may exhibit greater seismic attenuation, possibly related to the greater presence of siliciclastic components or internal microstructures that promote wave dispersion mechanisms. According to Han et al.,^37^ seismic energy dissipation in sedimentary rocks is directly associated with mineralogical content and porosity distribution, as the presence of minerals with different elastic moduli can amplify attenuation due to acoustic impedance contrast between grains.

The point with the highest porosity (∼18.5%) and an intermediate *Q*_p_ value (∼13) presents a dark blue coloration, indicating a very low calcite content (∼10%). This behavior reinforces the hypothesis that the mineralogical composition, combined with the porosity distribution, plays a fundamental role in seismic energy dissipation. As described by Anselmetti and Eberli,^38^ increased porosity reduces the matrix stiffness and consequently decreases the *Q*_p_ quality factor, as more porous structures facilitate mechanical energy dissipation through friction and fluid movement within the pores.

Figure 9 presents a scatter plot relating porosity (%) on the *x*-axis and the quality factor of P-waves (*Q*_p_) on the *y*-axis, with the color of the points representing permeability (mD), as indicated by the color scale on the right. The legend classifies the samples as grainstone or rudstone, according to the classification by Dunham,^31^ highlighting lithological differences that influence the acoustic and hydraulic properties of carbonate rocks.

**Figure 9:**
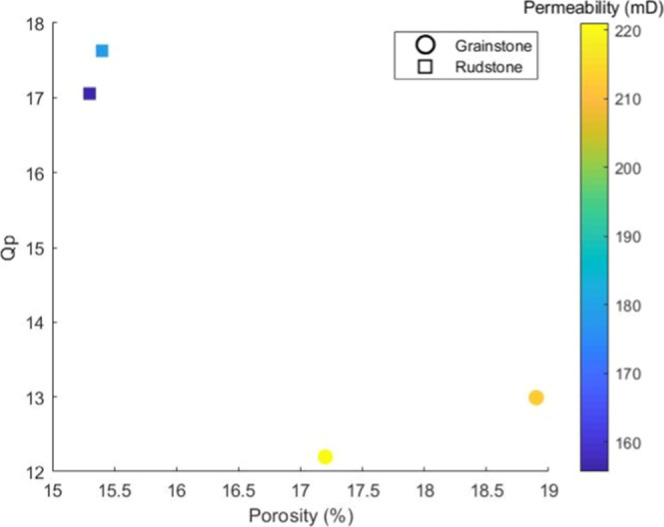
Scatter plot showing the relationship between porosity (%) on the *x*-axis and P-wave quality factor (*Q*_p_) on the *y*-axis. The color of each data point represents permeability (mD), as indicated by the color scale to the right. Samples are classified as grainstone or rudstone based on Dunham’s carbonate classification.

The samples classified as rudstone exhibit lower porosity (∼15.5%) and permeability values but display the highest *Q*_p_ values (∼17–18). This behavior suggests that such rocks have a more compact and mechanically rigid matrix, resulting in lower seismic energy dissipation.

On the other hand, the samples classified as grainstone exhibit the highest porosity values (∼18.5%) and permeability values but present the lowest *Q*_p_ values (∼12–13). This pattern aligns with investigations demonstrating that rocks with higher porosity exhibit greater seismic dissipation, as the porous structure facilitates wave dispersion mechanisms such as friction and movement of interstitial fluids (Anselmetti and Eberli^38^ and Han et al.^37^).

The proximity of the samples classified as rudstone in the graph can be explained by their composition and depositional structure, which results in relatively homogeneous petrophysical properties within this group. According to Dunham,^31^ rudstones are carbonate rocks dominated by clasts larger than 2 mm, supported by a carbonate matrix or intergranular cementation. This structure tends to generate relatively low porosity as compaction and mineral matrix filling significantly reduce pore connectivity. Consequently, the permeability of these rocks is also limited, restricting fluid movement.

In the graph, the rudstone samples exhibit similar *Q*_p_ values (∼17–18), indicating that they have similar acoustic behavior. This occurs because the low porosity and high mechanical rigidity of the mineral matrix minimize seismic energy dissipation, as noted in studies on wave attenuation in carbonates (Carcione et al.^36^ and Eberli et al.^35^). The proximity of these samples suggests that internal lithological variations, such as the degree of cementation and grain arrangement, are not sufficient to generate significant dispersions in the *Q*_p_ values, confirming that their compact structure dominates the acoustic behavior.

On the other hand, the samples classified as grainstone also appear grouped but in a distinct region of the graph, characterized by higher porosity (∼17–19%) and lower *Q*_p_ (∼12–13). This occurs because grainstones, as defined by Dunham,^31^ are predominantly composed of grain-supported sediments with no significant matrix, favoring the existence of well-connected intergranular pores. This high pore connectivity results in greater permeability, allowing greater fluid flow and amplifying seismic wave dissipation mechanisms (Anselmetti and Eberli^38^).

The proximity of the grainstone samples suggests that despite possible variations in the grain size distribution and degree of cementation, the dominant porous structure results in a similar acoustic pattern among the samples. Studies indicate that rocks with high porosity and permeability exhibit greater dispersion of seismic energy due to fluid–rock interactions, which significantly reduces the quality factor of P-waves (Dvorkin and Nur^8^ and Han et al.^37^). Thus, the trend observed in the graph reinforces the expected relationships among the porosity, permeability, and seismic attenuation in heterogeneous carbonates.

Figure 10 presents a scatter plot relating porosity (%) on the *x*-axis and the P-wave quality factor (*Q*_p_) on the *y*-axis, with the color of the data points representing permeability (mD), as indicated by the color scale on the right. The legend identifies different types of porosity classified according to Lucia.^32^

**Figure 10:**
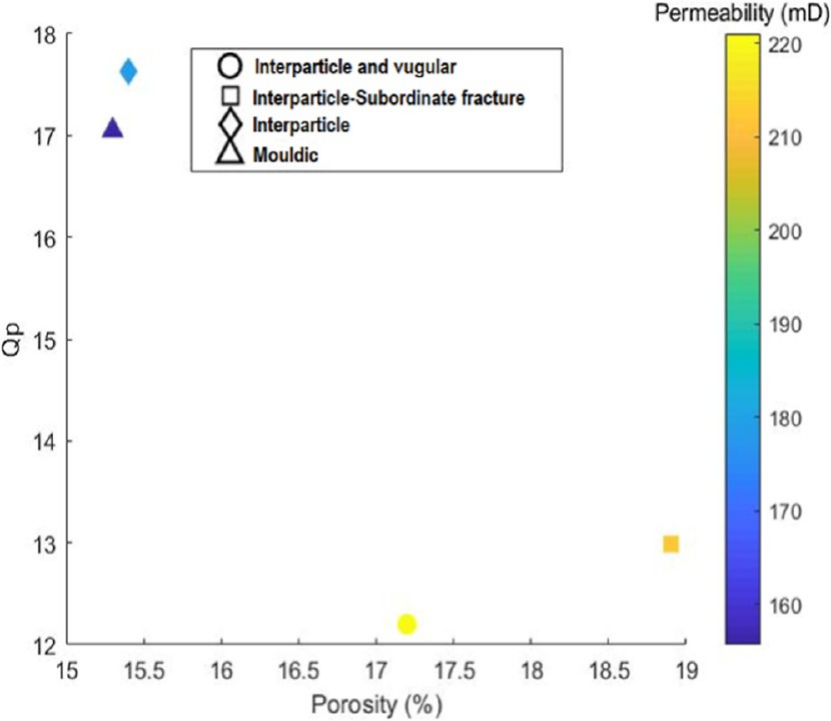
Scatter plot showing the relationship between porosity (%) on the *x*-axis and P-wave quality factor (*Q*_p_) on the *y*-axis. The color of each point represents permeability (mD), as indicated by the color scale on the right. Data points are categorized according to dominant pore type.

The samples with interparticle and vuggy porosities, as well as those with interparticle porosities associated with fractures, exhibited higher seismic attenuation values than did the samples classified with moldic and interparticle porosities. This behavior can be attributed to the greater pore connectivity and the presence of fractures, which enhance energy dissipation mechanisms such as fluid movement and grain friction, leading to increased P-wave attenuation.

Figure 11 presents the characteristic frequencies associated with the three main seismic attenuation mechanisms in porous rocks (coquinas) as a function of saltwater saturation (*S*_w_). The data correspond to four samples from the Morro do Chaves Formation (7D-AL, 6E-AL, 9C-AL, and 4.3B-AL), and the experimental measurement frequency was 1.3 MHz, as indicated by the red dashed line in the graph.

**Figure 11:**
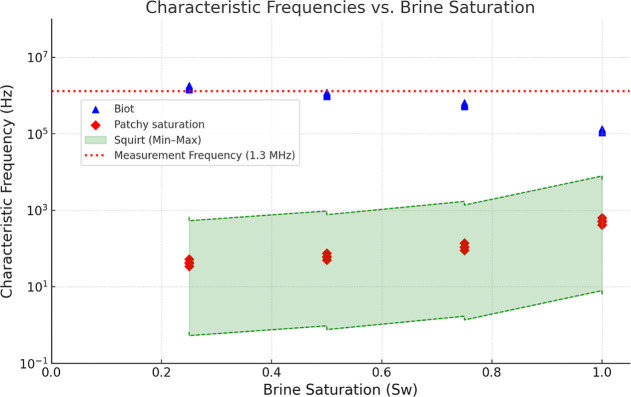
Characteristic frequencies of the main seismic attenuation mechanisms as a function of brine saturation (*S*_w_) for four carbonate rock samples (7D-AL, 6E-AL, 9C-AL, and 4.3B-AL). The *x*-axis shows brine saturation, while the *y*-axis displays characteristic frequencies on a logarithmic scale. Blue triangular markers indicate critical frequencies from the Biot high-frequency model; red diamond markers correspond to characteristic frequencies from the Patchy Saturation model. The shaded green band represents the estimated frequency range for the squirt flow mechanism, based on variations in pore aspect ratio. A red dashed line marks the experimental measurement frequency (1.3 MHz).

The mechanism with the characteristic frequencies closest to the measurement frequency is the Biot model, represented by blue triangular markers. Originally proposed by Biot^18^ and later refined by Mavko et al.,^5^ this model describes energy dissipation through relative fluid–solid motion within a porous medium. The critical frequency associated with this mechanism is directly influenced by properties, such as porosity, permeability, fluid viscosity, and fluid density. Across the entire saturation range, the Biot frequencies remain close to 1.3 MHz, suggesting that this is the predominant attenuation mechanism under the experimental conditions.

The second mechanism considered is squirt flow, represented in the graph by the shaded green band, which delimits the minimum and maximum characteristic frequencies estimated on the basis of the pore aspect ratio and the bulk modulus of the mineral matrix. Squirt flow is associated with local fluid redistribution between pores of different geometries under pressure variations and is highly sensitive to rock microstructure (Mavko et al.,^5^ Gurevich et al.,^19^ and Alkhimenkov and Quintal^20^). Although this mechanism is recognized as important in carbonate rocks, particularly those with significant microporosity, such as coquinhas, the results indicate that its characteristic frequencies are generally shifted relative to the measurement frequency: considerably lower at low *S*_w_ values and significantly higher at high saturations. This finding indicates that squirt flow is not dominant at the 1.3 MHz measurement frequency, although it may contribute marginally under intermediate saturation conditions.

The third mechanism analyzed is patchy saturation, which is modeled according to the formulation of White et al.^21^ and represented by red diamond markers in the graph. This mechanism accounts for dissipation associated with mesoscopic-scale saturation heterogeneity, which generates pore pressure gradients and induces fluid flow between adjacent regions with different saturation states. The characteristic frequencies predicted by this model fall well below the measurement frequency (typically between 30 and 600 Hz), making this mechanism irrelevant under the experimental conditions adopted. Nevertheless, it is included because of its conceptual importance and widespread discussion in studies involving wave propagation in partially saturated media (Pride et al.,^22^ Rubino et al.,^23^ Tisato and Quintal,^39^ and Chapman et al.^24^).

A graph was created to show the variation in the inverse of the *Q*_p_ factor versus saltwater saturation (*S*_w_) (Figure 12). Two distinct groupings can be observed: one for samples 7D-AL and 6E-AL and the other for samples 9C-AL and 4.3B-AL. This grouping can be attributed to the similarities in porosity and permeability among these samples.

**Figure 12:**
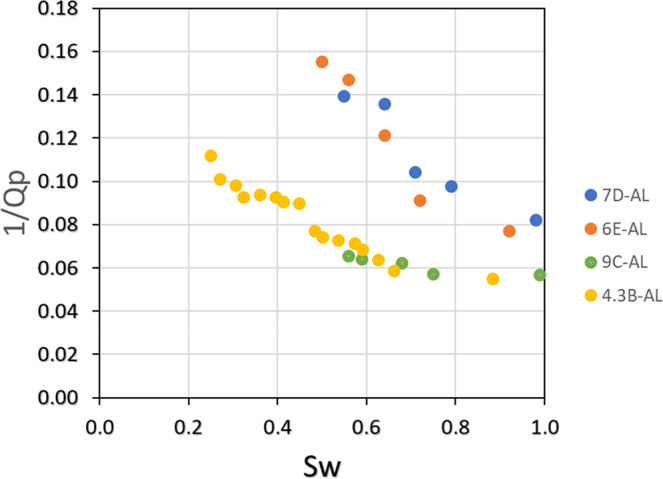
Graph of the P-wave quality factor as a function of oil saturation, with samples initially saturated with saltwater prior to oil injection.

The data presented in Figure 13 can be fitted to a potential relationship, as illustrated in eq 19. All regressions demonstrated a high coefficient of determination (*R* ^2^ > 0.8). Therefore, the relationship between 1/*Q*_p_ and *S*_w_ can be expressed as follows:

19 $$\frac{1}{Q_{p}}=a\cdot {S_{w}}^{-b}$$

**Figure 13:**
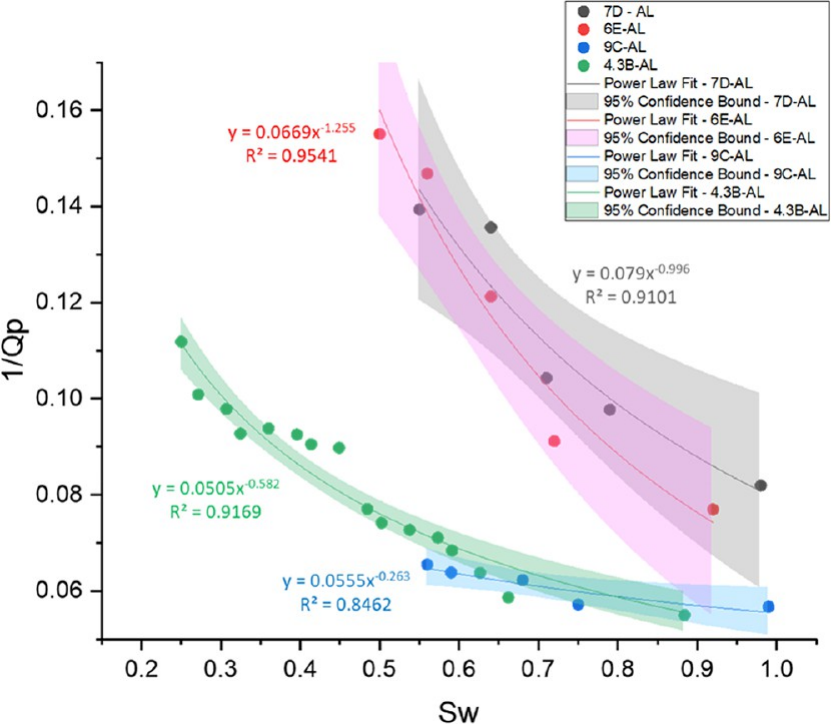
Relationship between brine saturation (*S*_w_) and inverse P-wave quality factor (1/*Q*_p_). Each data set is fitted with a power-law regression model, and the respective equations and *R* ^2^ values are shown in the same color as the data. Shaded bands represent the 95% confidence intervals of the fitted curves.

Upon examination of the clusters and coefficients from the potential adjustments, a correlation can be observed between these coefficients and the ratio of porosity to permeability (ϕ/*k*). Figure 14 shows a polynomial relationship between coefficient a and the porosity. Similarly, Figure 15 shows a polynomial correlation between coefficient b and the ratio of porosity to the logarithm of permeability.

**Figure 14:**
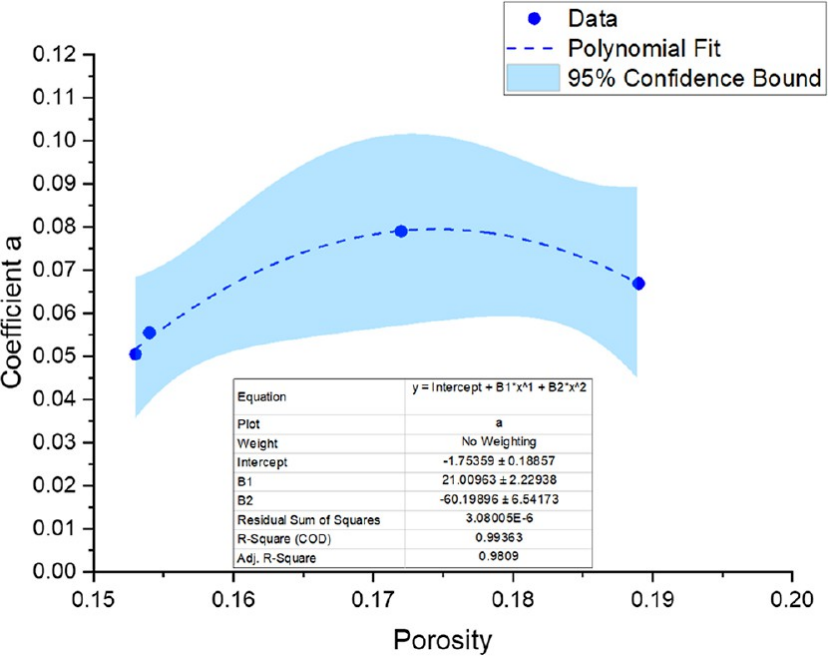
Polynomial regression (second-order) applied to the data set, showing the relationship between coefficient *a* and porosity. The dashed blue line represents the fitted curve, while the shaded area corresponds to the 95% confidence interval. The regression equation, coefficients, and statistical parameters (*R* ^2^, adjusted *R* ^2^, and residual sum of squares) are summarized in the embedded table.

**Figure 15:**
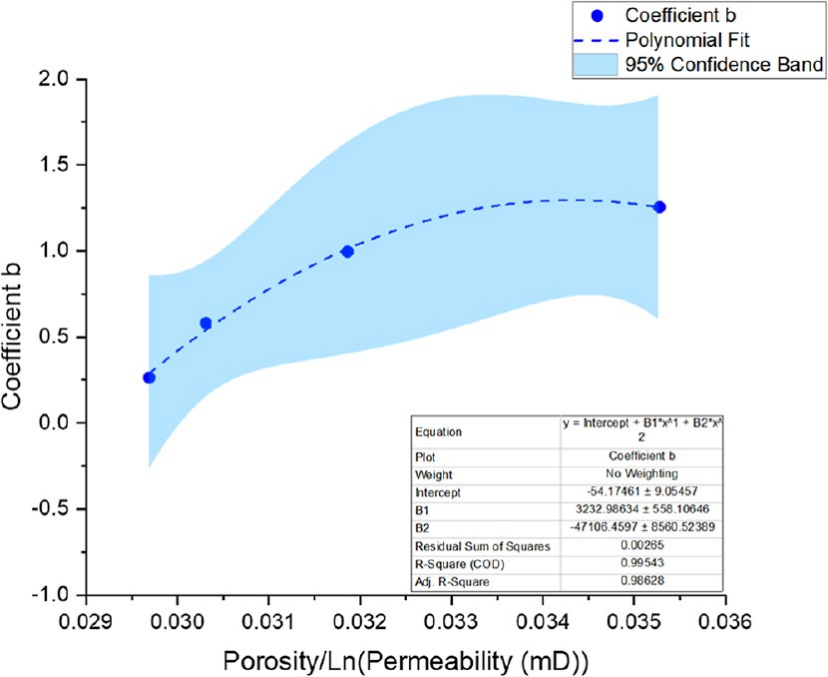
Polynomial regression (second-order) applied to the data set, showing the relationship between coefficient *b* and the ratio of porosity to the logarithm of permeability. The dashed blue line represents the fitted curve, while the shaded area corresponds to the 95% confidence interval. The regression equation, coefficients, and statistical parameters (*R* ^2^, adjusted *R* ^2^, and residual sum of squares) are summarized in the embedded table.

On the basis of the observations described above, an empirical model can be developed to fit the observed data, as represented by eqs 20 and 21.

20 $$b={-47,106\left(\frac{\Phi }{\ln\left(k\right)}\right)}^{2}+3233\left(\frac{\Phi }{\ln\left(k\right)}\right)-54.175$$

21 $$a=-60.199\Phi^{2}+21.01\Phi -1.7536$$

The results of this model are presented in Figure 16, which demonstrates a coefficient of determination (*R* ^2^) of 0.96.

**Figure 16:**
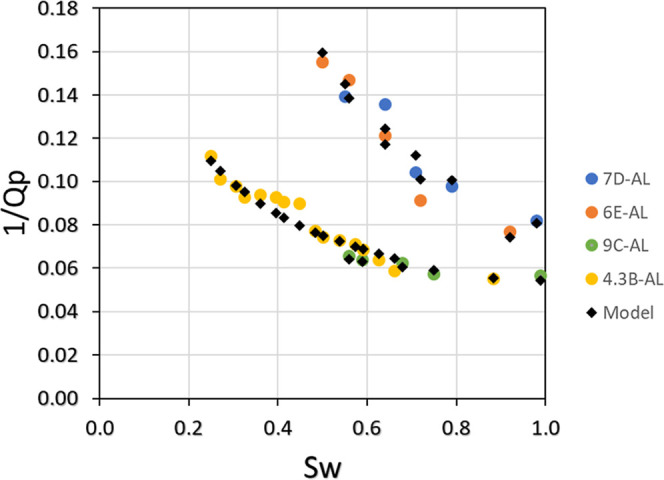
Comparison between the empirical model results (represented by black diamonds) and the observed data (depicted as colored circles).

The regression models presented in Figures 13–16 were selected on the basis of their strong statistical performance, as evidenced by high coefficients of determination (*R* ^2^ > 0.8). However, importantly, a good mathematical fit does not inherently imply a physical generalizability. These empirical relationships were derived under controlled laboratory conditions using ultrasonic frequencies, fixed effective pressures, and carbonate rock samples (coquinas) with complex pore architectures.

Consequently, caution must be exercised when attempting to extrapolate these models to the field scale, especially within the seismic frequency range (10–100 Hz). Seismic attenuation is a frequency-dependent phenomenon, and the dominant dissipation mechanisms in laboratory experiments (e.g., squirt flow) may differ from those active at the reservoir scale (e.g., Biot-type relaxations and fluid pressure equilibration between pore spaces).

While the trends observed offer meaningful insight into the relationship between attenuation and saltwater saturation in controlled environments, further studies are necessary to evaluate their applicability under actual reservoir conditions. Future research should focus on integrating these empirical results with frequency-dependent rock physics models that can bridge laboratory and field scales, thereby enhancing the predictive power of attenuation-based reservoir characterization techniques.

## Conclusions

This study comprehensively investigated the behavior of compressional wave attenuation in carbonate rocks during fluid substitution, utilizing coquina samples from the Morro do Chaves Formation, recognized as a representative analogue of Brazilian pre-salt carbonate reservoirs. Through laboratory experiments conducted at an ultrasonic frequency of 1.3 MHz and employing the spectral ratio method, it was possible to quantify the inverse quality factor (1/*Q*_p_) as a function of saltwater saturation (*S*_w_), considering four samples with varying textures, porosities, and permeabilities.

The results revealed a consistent trend of increased attenuation (1/*Q*_p_) as saltwater was progressively replaced by oil, indicating greater energy dissipation in environments with a lower water saturation. This variation is attributed to the intensification of dissipation mechanisms related to the fluid mobility within the porous matrix. Among the theoretical models considered, Biot’s attenuation mechanism proved to be the most representative under the experimental conditions adopted, accounting for the majority of energy dissipation observed in the analyzed samples. This mechanism, involving the decoupling between the solid matrix and the pore fluid, is particularly sensitive to the elastic properties of the rock and fluid viscosity, aligning with the frequency range utilized and the characteristics of the Coquinas from the Morro do Chaves Formation.

The complex pore structure of these rocks, including moldic, vuggy, interparticle, and interparticle with fracture porosities, significantly contributed to the attenuation response, confirming that the pore architecture plays a fundamental role in dissipation mechanisms. The adjusted empirical models exhibited high correlation coefficients (*R* ^2^ > 0.8), demonstrating the reliability of the observed trends between attenuation and saturation.

Although the measurements were conducted at ultrasonic frequencies, the results provide relevant insights into the frequency dependence of attenuation and the influence of fluid mobility factors of direct interest for field-scale seismic applications. The scarcity of experimental studies involving fluid substitution in heterogeneous carbonate rocks underscores the originality of this work. The findings presented herein contribute significantly to the advancement of rock physics modeling and the incorporation of attenuation attributes in the seismic interpretation of complex reservoirs.

## References

[1] Burchette T. P. (2012). Carbonate
rocks and petroleum reservoirs: a geological perspective from the
industry. Geol. Soc. Spec. Publ..

[2] Prasad M. (2003). Velocity-permeability
relations within hydraulic units. Geophysics.

[3] Hollis C., Vahrenkamp V., Tull S., Mookerjee A., Taberner C., Huang Y. (2010). Pore system characterisation in heterogeneous
carbonates: an alternative approach to widely-used rock-typing methodologies. Mar. Pet. Geol..

[4] de
Castro D. D., da Rocha P. L. F. (2013). Quantitative parameters of pore types
in carbonate rocks. Braz. J. Geophys..

[5] Mavko, G. ; Mukerji, T. ; Dvorkin, J. The Rock Physics Handbook: Tools for Seismic Analysis of Porous Media; Cambridge University Press: New York, 2009.

[6] Toms J., Müller T. M., Gurevich B. (2007). Seismic attenuation in porous rocks
with random patchy saturation. Geophys. Prospect..

[7] Carcione J. M., Picotti S., Gei D., Avseth P. (2003). Gas detection and characterization
through rock-physics models and seismic forward modeling. Geophysics.

[8] Dvorkin J., Nur A. (1996). Elasticity of high-porosity sandstones: theory for two north sea
data sets. Geophysics.

[9] Winkler, K. ; Nur, A. Attenuation and velocity in dry and water-saturated Massillon sandstone. Expanded Abstracts of the 48th Annual International SEG Meeting; Society of Exploration Geophysicists: San Francisco, 1978; pp 302–305.

[10] Murphy, W. F. Effects of Microstructure and Pore Fluids on the Acoustic Properties of Granular Sedimentary Materials. Ph.D. Dissertation, Stanford University, Stanford, CA, 1982.

[11] Winkler, K. W. ; Murphy, W. F. Acoustic velocity and attenuation in porous rocks. In Rock Physics & Phase Relations: A Handbook of Physical Constants; Ahrens, T. J. , Ed.; Wiley: New York, 1995; pp 20–34.

[12] Zambuja N. C., Soares A. C. P., Holanda M. E. S., Rabelo F. R. P. (1998). Petrophysical
study of the rocks from the Morro do Chaves Formation (Sergipe Group,
Lower Cretaceous), Sergipe-Alagoas Basin. Bol.
Geociênc. Petrobras.

[13] Sousa, M. O. Microporosity in coquinas of the Morro do Chaves Formation, Alagoas Basin, NE Brazil. AAPG Annual Convention; American Association of Petroleum Geologists: Houston, 2006.

[14] Dvorkin, J. ; Mavko, G. Seismic wave attenuation in porous rocks: theoretical models and experimental data. SEG Technical Program Expanded Abstracts; Society of Exploration Geophysicists: Houston, 2006; pp 1850–1854.

[15] Deng, W. Mechanisms and Models of Seismic Attenuation. Tesis, University of Saskatchewan, Saskatoon, 2017.

[16] Gautam, K. Fluid Effects on Attenuation and Dispersion of Elastic Waves. Dissertation, Master in Geophysics, Faculty of Colorado, Colorado, United States, 2003.

[17] Schön, J. H. Physical Properties of Rocks: A Workbook; Elsevier: Amsterdam, 2011.

[18] Biot M. A. (1956). Theory
of propagation of elastic waves in a fluid-saturated porous solid.
II. Higher frequency range. J. Acoust. Soc.
Am..

[19] Gurevich B., Makarynska D., De Paula O. B., Pervukhina M. (2010). A simple model
for squirt-flow dispersion and attenuation in fluid-saturated granular
rocks. Geophysics.

[20] Alkhimenkov Y., Quintal B. (2024). A simple and accurate model for attenuation and dispersion
caused by squirt flow in isotropic porous rocks. Geophysics.

[21] White J. E., Mihailova N., Lyakhovitsky F. (1975). Low-frequency seismic waves in fluid-saturated
layered rocks. J. Acoust. Soc. Am..

[22] Pride S. R., Berryman J. G., Harris J. M. (2004). Seismic attenuation due to wave-induced
flow. J. Geophys. Res.:Solid Earth.

[23] Rubino J. G., Müller T. M., Milani M. (2011). Seismic attenuation and dispersion
due to wave-induced flow in porous rocks with double porosity. J. Geophys. Res.:Solid Earth.

[24] Chapman S., Tisato N., Quintal B., Holliger K. (2016). Seismic attenuation
in partially saturated Berea sandstone submitted to a range of confining
pressures. J. Geophys. Res.:Solid Earth.

[25] API Recommend Practices for Core Analysis. RP-40; American Petroleum Institute: Washington, DC, 1998.

[26] Albers E., Bakker B. M., Gustafsson L. (2002). Modeling response of glycolysis in
S. cerevisiae cells harvested at diauxic shift. Mol. Biol. Rep..

[27] Filho, N. C. A. ; Arienti, L. M. ; Cruz, F. E. G. Guidebook to the rift-drift Sergipe-Alagoas passive margin basin, Brazil. AAPG International Conference & Exhibition; American Association of Petroleum Geologists: Rio de Janeiro, 1998, pp 1–113.

[28] Toksöz M. N., Johnston D. H., Timur A. (1979). Attenuation of seismic waves in dry
and saturated rocks: I. Laboratory measurements. Geophysics.

[29] Marques, Z. M. Characterization of the attenuation coefficient of elastic waves in sedimentary rocks. Dissertation (Master’s in Civil Engineering), Federal University of Rio de Janeiro, Rio de Janeiro, 2009.

[30] Choquette P. W., Pray L. C. (1970). Geologic nomenclature and classification of porosity
in sedimentary carbonates. AAPG Bull..

[31] Dunham, R. J. Classification of carbonate rocks according to depositional texture. In Classification of Carbonate Rocks—A Symposium; Ham, W. E. , Ed.; American Association of Petroleum Geologists: Tulsa, 1962, pp 108–121.

[32] Lucia F. J. (1995). Rock-fabric/petrophysical
classification of carbonate pore space for reservoir characterization. AAPG Bull..

[33] ASTM International Standard Test Method for Laboratory Determination of Pulse Velocities and Ultrasonic Elastic Constants of Rock. ASTM D2845-08; ASTM International: West Conshohocken, 2005.

[34] Lima
Neto I. A., Misságia R. M., Ceia M. A., Archilha N. L., Hollis C. (2015). Evaluation of carbonate pore system under texture control
for prediction of microporosity aspect ratio and shear wave velocity. Sediment. Geol..

[35] Eberli G. P., Baechle G. T., Anselmetti F. S., Incze M. L. (2003). Factors controlling
elastic properties in carbonate sediments and rocks. Leading Edge.

[36] Carcione J. M., Picotti S., Santos J. E. (2012). Numerical
experiments of fracture-induced
velocity and attenuation anisotropy. Geophys.
J. Int..

[37] Han D. H., Nur A., Morgan D. (1986). Effects of
porosity and clay content on wave velocities
in sandstones. Geophysics.

[38] Anselmetti F. S., Eberli G. P. (1999). The velocity-deviation log: a tool to predict pore
type and permeability trends in carbonate drill holes from sonic and
porosity or density logs. AAPG Bull..

[39] Tisato N., Quintal B. (2013). Measurements of seismic
attenuation and transient fluid
pressure in partially saturated Berea sandstone: evidence of fluid
flow on the mesoscopic scale. Geophys. J. Int..

